# Combined laparoscopic and thoracoscopic approach for tension gastrothorax in a foramen of Bochdalek hernia

**DOI:** 10.1016/j.ijscr.2019.10.076

**Published:** 2019-11-02

**Authors:** Saurabh Gandhi, Ajay Bhandarwar, Nidhisha Sadhwani, Chintan Patel, Amol Wagh, Eham Arora

**Affiliations:** Department of General Surgery, Grant Government Medical College & Sir JJ Group of Hospitals, Mumbai, India

**Keywords:** Tension gastrothorax, Borchardt’s triad, Gastric volvulus, Bochdalek hernia, Laparoscopy, Thoracoscopy

## Abstract

**Introduction:**

The incidence of Bochdalek hernias in adults is much more than previously reported. Though most of these are asymptomatic. Tension gastrothorax is a rare complication of these. Literature about the endoscopic management of a tension gastrothorax is extremely sparse. In this case report, we describe how we combined laparoscopy and thoracoscopy in an emergency setting to manage a case of tension gastrothorax with an underlying Bochdalek hernia defect in an adult.

**Patient profile:**

A 27 year old male presented to our emergency with tension gastrothorax and a gastric volvulus with an underlying Bochdalek hernia defect, exhibiting the classical Borchardt’s triad. The patient underwent an emergency surgery, through a minimally invasive approach wherein the herniated contents were reduced, the gastric volvulus detorted and a repair of the diaphragmatic defect was performed. His post-operative course was uneventful.

**Discussion:**

Tension gastrothorax, is a diagnostic challenge as an air-fluid level in the thorax on radiology with worsening respiration causes as many as 38% of these cases to be misdiagnosed as tension pneumothorax, hydro-pneumothorax, hemothorax, empyema, effusion or pneumonia. Various techniques of decompression have been described in literature but, employing those, in case of a misdiagnosis may have catastrophic outcomes.

**Conclusion:**

We strongly recommend employing a combined laparoscopic and thoracoscopic approach for an emergency repair of a tension gastrothorax in a hemodynamically stable patient as, it poses all the advantages of minimal access surgery and is available, at smaller centres, even in an emergency.

## Highlights

• Combined laparoscopy and thoracospy to be used in tension gastrothorax.
• Minimally invasive approach to be used even in an emergency.
• Invasive pre-operative decompression to be avoided.

## 1 Introduction

The incidence of Bochdalek hernias in adults is much more than previously reported. The previously reported incidence of was 0.17%, erring on the conservative side. Though most of these are asymptomatic, symptoms and complications occur due to ventilatory compromise or luminal compression of abdominal viscera. Tension gastrothorax is a rare complication which is difficult to diagnose. An air-fluid level in the thorax on radiology with worsening respiration causes as many as 38% of these cases to be misdiagnosed as tension pneumothorax, hydro-pneumothorax, hemothorax, empyema, effusion or pneumonia [1]. Our literature search revealed only a handful of cases [[2], [3], [4]] of tension gastrothorax with none of them being managed endoscopically. Our work has been reported in line with the SCARE criteria.

## 2 Case scenario

### 2.1 Patient profile

A 27 year old gentleman presented to the emergency department at our institute, with a gradually worsening epigastric pain and retching without vomiting for one day. He had been breathless for two hours, without any precipitating trauma or history of similar episodes.

On examination, he was anxious, tachycardic (136/minute), hypotensive (88/60 mm Hg) with an oxygen saturation of 84% in room air. His neck veins were visibly distended. He had a hyper-resonant note in the left hemithorax on percussion with absent breath sounds on auscultation with epigastric tenderness. Attempts to pass a naso-gastric tube were met with stiff resistance 30 cm from incisors.

**Fig. 1:**
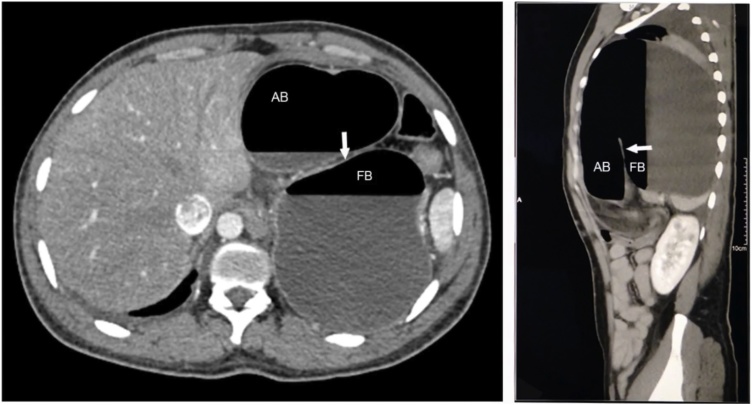
CT showing the gastric volvulus.

**Fig. 2:**
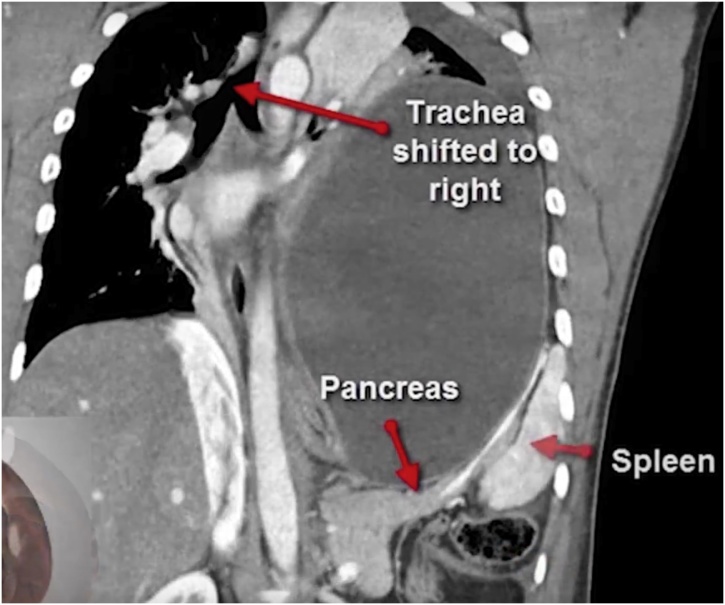
CT showing intra-thoracic transverse colon and pancreas.

His chest radiographs showed a large air-fluid level in his left hemithorax, making a hydropneumothorax a probable diagnosis. The epigastric tenderness and inability to pass a naso-gastric tube were inconsistent with this diagnosis, which prompted a CT. The scan showed a vastly dilated stomach with a transition line between the antral and fundic air bubbles due to a volvulus (Fig. 1). The stomach, spleen, distal pancreas and transverse colon were within the left thoracic cavity, passing through a postero-lateral diaphragmatic defect measuring 6 × 6.7 cm, with resultant compression of the ipsilateral lung and a rightward mediastinal shift (Fig. 2). This confirmed a diagnosis of tension gastrothorax with a gastric volvulus within a Bochdalek hernia. The patient was hemodynamically optimised, correcting dehydration, and taken up for emergent repair.

### 2.2 Operative procedure

**Fig. 3:**
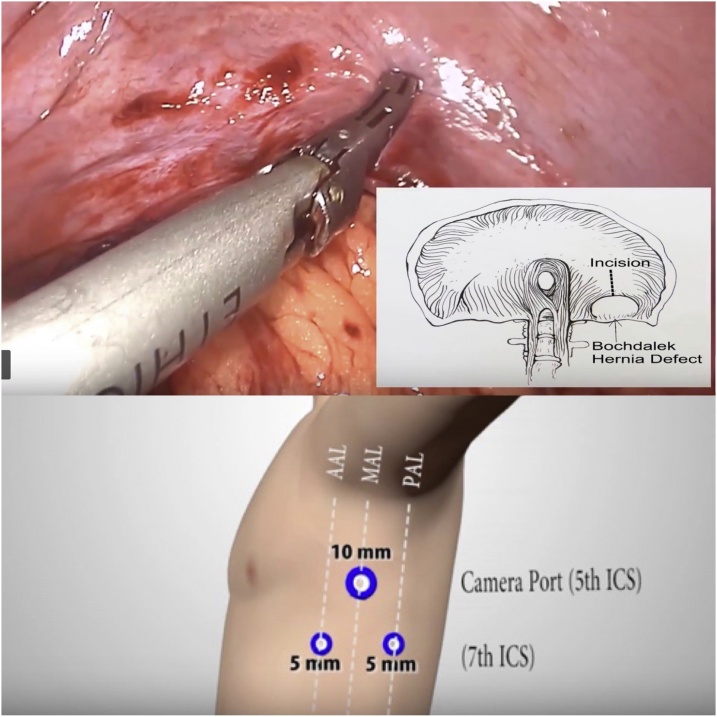
Pendulum shaped incision over the diaphragm (upper), thoracoscopic port placement (lower).

**Fig. 4:**
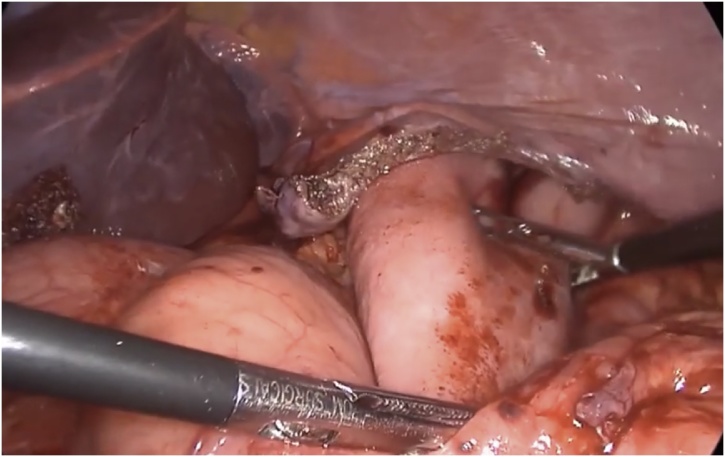
Drawing the stomach out of the diaphragmatic defect.

**Fig. 5:**
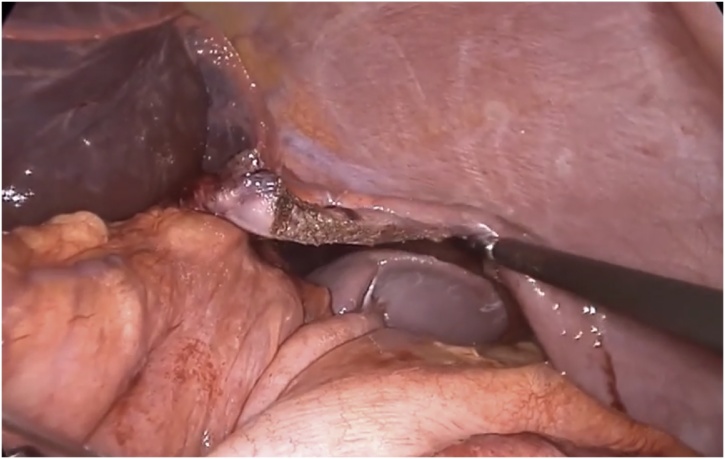
Thoracic location of the spleen as visualised transabdominally through the defect after replacing the stomach in the abdomen.

**Fig. 6:**
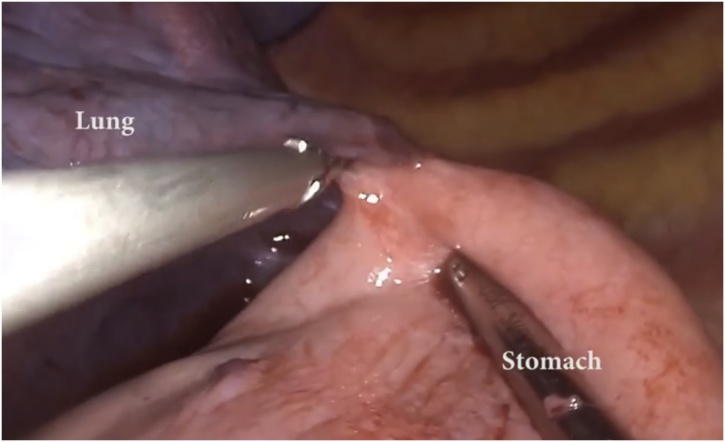
Adhesions between the lung and the stomach in the thorax.

**Fig. 7:**
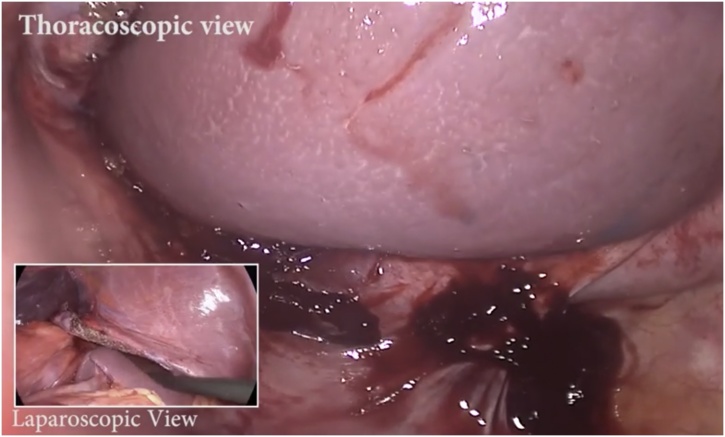
Combined thoracosopic and laparoscopic views of the spleen.

**Fig. 8:**
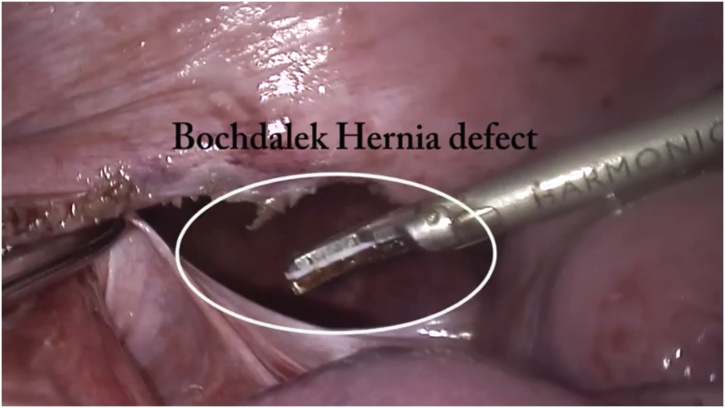
Bochdalek hernia defect.

General anesthesia via single-lung ventilation was employed. With the patient in right lateral position, ports were placed. A diagnostic laparoscopy revealed a stretched left hemidiaphragm with convexity towards the abdomen. The stomach, spleen and left segment of transverse colon were missing, with a stretch on their vascular attachments. With an aim to reduce contents back into the abdomen, dissection commenced at the medial end of the posterolateral diaphragmatic defect. The omentum was reduced into the peritoneal cavity and adhesions to the colon were divided with ultrasonic shears. Due to an overdistended stomach, we were unable to reduce hollow viscera through the native defect. The defect was widened with a 2 cm incision medially, resembling a pendulum arm (Fig. 3). This facilitated reduction of the stomach under gentle traction (Fig. 4). At this point, a naso-gastric tube was passed sans resistance, confirming derotation of gastric volvulus, permitting suction of luminal content. Minimal pleural fluid collected within the thorax was suctioned through the diaphragmatic defect. Close apposition of the abdominal viscera with the lung necessitated prudent use of energy with most dissection progressing in blunt fashion (Figs. 5, 6). At the end of adhesiolysis with reduction of all viscera (Fig. 7), the posterior lip of diaphragmatic defect was seen to consist of only a narrow rim of muscle and peritoneum (Fig. 8).

**Fig. 9:**
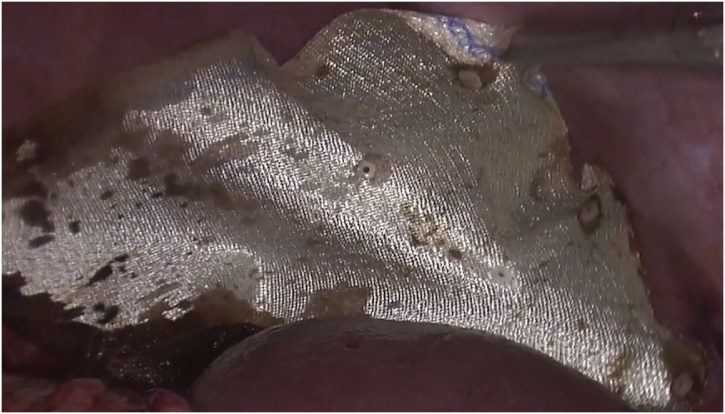
15 × 15 bilaminar mesh placement.

The abdominal viscera, particularly the spleen and stomach, tended to pull back into the thoracic cavity after reduction. This “memory” of herniated viscera combined with difficulty reaching the posterior edge of the defect prompted us to close the defect thoracoscopically. Isolated right-lung ventilation greatly aided the insertion of ports as depicted in Fig. 3. The laparoscopic and thoracoscopic teams worked in tandem, with the former retracting viscera into the abdomen while the latter closed the defect with continuous polypropylene sutures. The laparoscopic team augmented defect closure by placing a 15 × 15 cm bilaminar mesh over the defect, fixing it to the diaphragm with interrupted nonabsorbable sutures (Fig. 9). At this point, the left lung was ventilated and checked for air leaks. An intercostal under-water seal drain was introduced via the 10 mm port. The surgery ended with placement of an abdominal drain in the left upper quadrant.

**Fig. 10:**
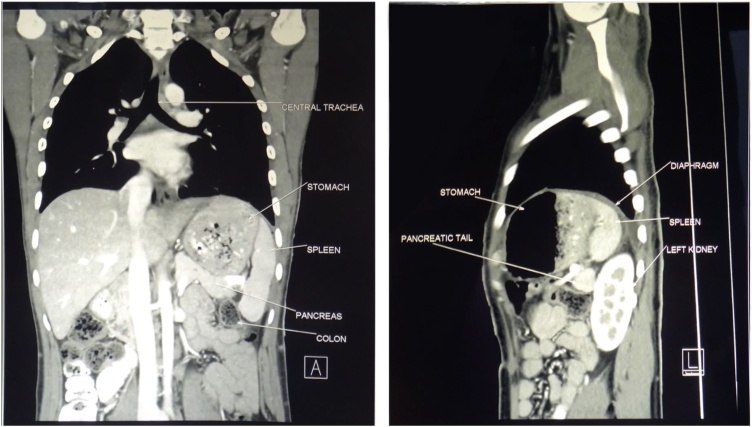
Post operative scan.

The interval between arrival at hospital and surgery was 90 min. The operative time was 160 min. The patient was cared for in an intensive care unit for 2 days without need of ventilatory support. A CT scan on the 2nd postoperative day showed full expansion of the left lung without any evidence of herniation (Fig. 10). He was discharged 4 days after surgery and suffered no clinical or radiological recurrence 15 months later.

## 3 Discussion

The term “tension” refers to worsening pressures due to unidirectional air flow into the pleural space, with rapid clinical deterioration. Tension gastrothorax differs in causative pathology with fairly similar consequences as tension pneumothorax. The stomach prolapses into the thorax through a congenital or acquired diaphragmatic defect with pulmonary compression and ventilatory compromise. Due to rotation of stomach about its axis, its inlet and outlet are obstructed forming a “closed loop”. However, the acutely angulated gastroesophageal junction can function as a one-way valve allowing swallowed secretions and air to pass into the stomach causing progressively worsening distension. Like tension pneumothorax, gastrothorax causes worsening mediastinal and contralateral lung compression, which can culminate in cardiac arrest. This pathology was first described by Ordog et al. in 1984, in a case of traumatic rupture of the diaphragm [5]. Five steps are necessary to develop a tension gastrothorax: (1) existence of a diaphragmatic defect, (2) increased intra-abdominal pressure, (3) prolapse of the stomach into the thorax, (4) a functional change in the gastro-esophageal junction (by way of an abnormal angulation) and (5) a reduction in cardiac output as a result of mediastinal shift [6,7].

Abnormal rotation of the stomach by more than 180 degrees is unusual, due to its numerous attachments. In congenital diaphragmatic hernia, these gastric attachments are lax or absent, increasing the risk of volvulus [8]. Spontaneous diaphragmatic hernias are rare. Acquired diaphragmatic hernias often follow blunt or penetrating trauma, including surgery (tube thoracostomy, laparoscopic cholecystectomy, hepatectomy, gastric banding, and plication for diaphragmatic eventration).

Symptomatic Bochdalek’s hernias are rare in adults and acute tension gastrothorax, even more so. Due to its scarce occurrence, tension gastrothorax with an underlying Bochdalek hernia is not amongst the top differential diagnoses of a patient with respiratory distress and an air-fluid level on X-ray. Hydropneumothorax or tension pneumothorax are more likely, but hasty insertion of a chest tube in a misdiagnosed case can lead to hollow viscus or vascular injury with catastrophic outcomes. The clues in our patient were epigastric tenderness and an inability to perform naso-gastric intubation. The experience of our center in dealing with large diaphragmatic hernias further aided diagnosis. A chest X-ray improves diagnostic accuracy and one showing the tip of naso-gastric tube in an intrathoracic stomach or within the lower esophagus will confirm the diagnosis. But, a CT with coronal and sagittal images is the most useful form of imaging.

Our case exhibited all three features of acute gastric volvulus - frequent unproductive retching, severe abdominal pain and inability to insert a nasogastric tube, originally described by Borchardt (Borchardt’s triad) [9]. When untreated, patients can develop gastric ischemia, perforation, acute haemorrhage, pancreatic necrosis and omental avulsion. Management involves preoperative nasogastric decompression during fluid resuscitation. When successful, nasogastric decompression produces instant clinical improvement. When available, emergency endoscopic decompression is a good option. Previously performed manoeuvres such as percutaneous aspiration of gastric contents and trocar-assisted chest drain insertion are now obsolete as they significantly increase patient morbidity and preclude the use of an exogenous prosthesis due to the contamination of the thoracic cavity by gastric contents [10,11]. Once resuscitated, emergency surgical treatment includes derotation of volvulus, reduction of the herniated contents, closure of the diaphragmatic defect and gastropexy to the anterior abdominal wall. Laparoscopy, thoracoscopy, or a combination of both can be used. Laparoscopy has the added advantage of surveying the abdomen for trauma to abdominal viscera, while thoracoscopic repair of diaphragm can be ergonomically simpler.

## 4 Conclusion

Tension gastrothorax with an intra thoracic gastric volvulus, is a life threatening condition which can rapidly result in fatal cardiorespiratory distress and a cardiac arrest. Early diagnosis and surgery is extremely crucial in these cases. We strongly recommend employing a combined laparoscopic and thoracoscopic approach for an emergency repair in a young, hemodynamically stable patient.

## Declaration of Competing Interest

None.

## Sources of funding

None.

## Ethical approval

Our study is exempt from ethical approval.

## Consent

The patient consent has been obtained and will be sent to the Editor-in-Chief.

## Author contribution

Dr. Saurabh Gandhi helped draft the operative section of the manuscript and performed literature review.

Dr. Ajay Bhandarwar was the lead surgeon on the case, helped draft the manuscript and reviewed it prior to submission.

Dr. Nidhisha Sadhwani helped draft the manuscript discussion, edited images and is the corresponding author.

Dr. Chintan Patel helped perform literature review and helped condense the manuscript to meet author guidelines.

Dr. Amol Wagh was assistant operating surgeon and performed a literature review.

Dr. Eham Arora helped draft the manuscript.

## Registration of research studies

Not applicable.

## Guarantor

Dr. Ajay Bhandarwar.

## Provenance and peer review

Not commissioned, externally peer-reviewed.
